# Ratio of Electron Donor to Acceptor Influences Metabolic Specialization and Denitrification Dynamics in *Pseudomonas aeruginosa* in a Mixed Carbon Medium

**DOI:** 10.3389/fmicb.2021.711073

**Published:** 2021-09-10

**Authors:** Irene H. Zhang, Susan Mullen, Davide Ciccarese, Diana Dumit, Donald E. Martocello, Masanori Toyofuku, Nobuhiko Nomura, Steven Smriga, Andrew R. Babbin

**Affiliations:** ^1^Department of Earth, Atmospheric and Planetary Sciences, Massachusetts Institute of Technology, Cambridge, MA, United States; ^2^Program in Microbiology, Massachusetts Institute of Technology, Cambridge, MA, United States; ^3^Department of Marine Chemistry and Geochemistry, Woods Hole Oceanographic Institution, Woods Hole, MA, United States; ^4^Faculty of Life and Environmental Sciences, Microbiology Research Center for Sustainability, University of Tsukuba, Tsukuba, Japan

**Keywords:** *Pseudomonas aeruginosa*, denitrification, rate-yield tradeoff, specialization, nitrite

## Abstract

Denitrifying microbes sequentially reduce nitrate (NO_3_^–^) to nitrite (NO_2_^–^), NO, N_2_O, and N_2_ through enzymes encoded by *nar*, *nir*, *nor*, and *nos*. Some denitrifiers maintain the whole four-gene pathway, but others possess partial pathways. Partial denitrifiers may evolve through metabolic specialization whereas complete denitrifiers may adapt toward greater metabolic flexibility in nitrogen oxide (NO_x_^–^) utilization. Both exist within natural environments, but we lack an understanding of selective pressures driving the evolution toward each lifestyle. Here we investigate differences in growth rate, growth yield, denitrification dynamics, and the extent of intermediate metabolite accumulation under varying nutrient conditions between the model complete denitrifier *Pseudomonas aeruginosa* and a community of engineered specialists with deletions in the denitrification genes *nar* or *nir*. Our results in a mixed carbon medium indicate a growth rate vs. yield tradeoff between complete and partial denitrifiers, which varies with total nutrient availability and ratios of organic carbon to NO_x_^–^. We found that the cultures of both complete and partial denitrifiers accumulated nitrite and that the metabolic lifestyle coupled with nutrient conditions are responsible for the extent of nitrite accumulation.

## Introduction

Microbial assemblages in natural environments perform diverse biogeochemical transformations that drive global nutrient cycling and serve key ecological functions (Flemming and Wuertz, 2019). Among these, denitrification is a generally microbially mediated process that balances the nitrogen budget in terrestrial and marine ecosystems (Arrigo, 2005). Denitrifying microbes use nitrogen oxides (NO_x_^–^) as terminal electron acceptors under oxygen-limiting conditions, sequentially reducing in turn nitrate (NO_3_^–^) to nitrite (NO_2_^–^), nitric oxide (NO), nitrous oxide (N_2_O), and finally N_2_ through reductase enzymes encoded by the genes *nar* or *nap*, *nir*, *nor*, and *nos*, respectively (Zumft, 1997). As each step of denitrification yields free energy for the cell by coupling the reduction of nitrogen species to the oxidation of carbon, microbes theoretically harness the most energy for growth by performing the entire pathway. However, molecular surveys have revealed that many denitrifiers possess only partial denitrifying potential, whereas others contain the full suite of four genes (Zumft, 1997; Graf et al., 2014; Marchant et al., 2018). In addition, the polyphyletic distribution of denitrifying capabilities across diverse taxonomic groups and unique evolutionary history of each denitrification gene indicate the independent loss, gain, or horizontal transfer of these genes between microbes (Jones et al., 2008). Selective pressure to minimize the metabolic costs of enzyme biosynthesis, along with genome streamlining, may lead to the loss of individual denitrification genes (Mira et al., 2001; Giovannoni, 2005). Horizontal transfer may lead to the acquisition of genes that confer the ability to reduce available forms of inorganic nitrogen (Jones et al., 2008; Alvarez et al., 2011).

The modularity of denitrification genes, whether as a cause or function of the fragmentation of the denitrification pathway, points to possible metabolic specialization within these communities (Lycus et al., 2018). The phenomenon of metabolic specialization in microbial communities has been well-established (Johnson et al., 2012; Wong et al., 2015; D’Souza et al., 2018; Thommes et al., 2019; Meijer et al., 2020). Specialization may manifest as members of coexisting populations diversify to fill available niches defined by nutrient availability, spatial structure, temporal variability, or other factors. Laboratory experiments with model denitrifying organisms have determined that different genes involved in the denitrification process activate under distinct environmental cues and display unique dynamics (Lycus et al., 2018). Within denitrifying ecosystems, the availability of multiple inorganic nitrogen species may lead to the diversification of microbes into populations of NO_3_^–^ consumers (NO_2_^–^ producers) and NO_2_^–^ consumers. Specialization can also evolve if community members construct new niches through the release of metabolic byproducts which then become substrates for the growth of other members (Kinnersley et al., 2009; Lilja and Johnson, 2019). Additionally, partial denitrifiers may have evolved unique functions beyond canonical denitrification, such as detoxification by *nir* and *nor* of toxic chemical intermediates and cellular regulation and signaling using NO (Sasaki et al., 2016; Vázquez-Torres and Bäumler, 2016). As the reduction of NO_3_^–^ results in the production of the intermediate metabolites NO_2_^–^, NO, and N_2_O, which are released into the environment, NO_3_^–^-reducing microbes may create new metabolic niches for specialist populations that perform downstream denitrification steps. Over time, a community of complementary specialists relying on substrate cross-feeding of intermediate metabolites may arise.

The accumulation of intermediate metabolites may drive specialization by forming new ecological niches. Denitrification enzymes form dynamic, membrane-bound complexes *via* protein-protein interactions, which maximizes electron transfer efficiency (Borrero-de Acuña et al., 2017). Despite this tight relationship between denitrification proteins, intermediate metabolites, particularly NO_2_^–^, accumulate in both culture-based denitrification systems (Matsubara and Zumft, 1982; Granger and Ward, 2003; Bergaust et al., 2010) and in natural environments where denitrification occurs such as marine oxygen deficient zones (ODZs) (Brandhorst, 1959; Ulloa et al., 2012). This accumulation of metabolic intermediates may indicate a spatial separation of denitrification steps through partitioning different metabolic steps into separate cells or a temporal separation in the transcription of individual genes or the activity of individual enzymes.

A multitude of factors have been shown to influence the accumulation of intermediate denitrification metabolites. Previous studies indicate that lower organic carbon to NO_x_^–^ (C: NO_x_^–^) ratios result in significant NO_2_^–^ accumulation in an aquatic system (Chen et al., 2017). Other possible explanations invoke competition between denitrification enzymes for co-factors, membrane space, biosynthetic building blocks, or other intracellular resources (Almeida et al., 1995; Lilja and Johnson, 2016). The involvement of transporters may contribute to the accumulation of metabolites prior to movement across a membrane. Moreover, the metabolic costs of enzyme biosynthesis create a tradeoff between maintaining and activating the full denitrification pathway and specializing in only one or several steps (Pfeiffer and Bonhoeffer, 2004; Costa et al., 2006; Wortel et al., 2018). Minimizing biosynthesis costs in multi-enzyme pathways can lead to intermediate metabolite accumulation, giving rise to multiple specialist populations even upon a single resource (Treves et al., 1998; Pfeiffer and Bonhoeffer, 2004). Therefore, this tradeoff is a key element for the evolution and coexistence of species.

Complete denitrification may occur either through full reduction of NO_3_^–^ to N_2_ within one or several independent complete denitrifiers or as a community process between complementary partial denitrifiers. Here, we use laboratory cultures of model complete and partial denitrifiers to examine the tradeoffs involved in these two lifestyles and the effects of each lifestyle upon denitrification and growth dynamics. To eliminate the confounding factor of strain or species differences in comparing metabolic lifestyles, we use the wild-type complete denitrifier *Pseudomonas aeruginosa* and knockout strains with either a deletion in the gene for nitrite reductase (Δ*nir*) or a deletion in the gene for membrane-bound nitrate reductase (Δ*nar*), the respiratory nitrate reductase in canonical denitrification. *P. aeruginosa* occurs widely in marine, aquatic, and soil ecosystems and is attractive as a model organism due to its genetic tractability (Schreiber et al., 2007). We define the wild-type *P. aeruginosa* as a generalist in the context of denitrification, as it possesses the capability to utilize diverse oxidized nitrogen species as electron acceptors for energy. Conversely, we define the isogenic mutants as specialists since they possess only a defined subset of metabolic capabilities.

We compare the growth and denitrification dynamics of these two model specialists in co-culture against their parent wild-type *P. aeruginosa* under varying nutrient conditions. As environmental denitrifiers utilize heterogeneous organic carbon and inorganic nitrogen sources, we test four nutrient regimes characterized by differing ratios of mixed organic carbon to NO_x_^–^, specifically NO_3_^–^ and NO_2_^–^. We further use a varied organic medium with many compound classes more akin to natural systems than a medium with a single carbon source. Both total nutrient availability and carbon to nitrogen oxide ratios (C: NO_x_^–^) have been demonstrated to impact denitrification processes and the metabolic division of labor within communities (Blaszczyk, 1993; Ge et al., 2012; Chen et al., 2017), so we expect these regimes to exert different selective pressures on our model specialist vs. generalist communities, influencing their growth, denitrification dynamics, and accumulation of the intermediate NO_2_^–^.

## Materials and Methods

### Strains and Culture Methods

For the *Pseudomonas aeruginosa* Δ*nir* mutant, the region from *nirS* to *nirN* was deleted, while for Δ*nar* the *narG* gene was deleted (Toyofuku and Sawada, 2014). Isogenic mutants were constructed as follows: PCR primers listed in Supplementary Table 1 were used to amplify DNA fragments upstream and downstream of either *narG* or *nirS-N* with overlap extension PCR. The amplified fragments were cloned into a multicloning site in pG19II. The pG19-Δ*nar* or pG19-Δ*nir* plasmids were conjugated from *E. coli* S17-1 into wild-type *P. aeruginosa* PAO1 and deletion mutants were generated with allelic exchange. Deletions were confirmed with PCR and phenotypic analysis.

*Pseudomonas aeruginosa* wild-type PAO1 and mutant strains were inoculated into 25 mL of either 100% Luria-Bertani (LB) Broth (for regimes with 100% LB) or 10% LB Broth diluted with phosphate-buffered saline (PBS; for regimes with 10% LB). LB Broth was chosen due to its varied and complex carbon sources, which may more closely resemble conditions in natural systems preferred by heterotrophic bacteria in which various carbon sources derive from complex cellular metabolites. Through the use of LB, we hoped to avoid growth dynamics that depend on and are specific to the choice of an individual carbon compound. LB Broth also contains an abundance of reduced, organic nitrogen species for assimilatory anabolism, enabling supplemental NO_3_^–^ or NO_2_^–^ to be used primarily for dissimilatory energetic pathways. We additionally performed a control experiment in M9 minimal media supplemented with approximately 10 or 1 mM NO_3_^–^ and 50 or 5 mM citrate (a C_6_ compound) as the sole carbon source to confirm our results are specific to mixed carbon media. M9 minimal media contains 9.35 mM NH_4_ for nitrogen assimilation, allowing supplemental NO_x_^–^ to be used primarily for dissimilatory reduction. Additionally, M9 minimal media was supplemented with 4.1 nM biotin, 3.8 nM thiamin, 31 μM FeCl_3_, 6.2 μM ZnCl_2_, 0.76 μM CuCl_2_, 0.42 μM CoCl_2_, 1.62 μM H_3_BO_3_, and 0.081 μM MnCl_2_. Cultures were incubated overnight until reaching stationary phase at 37°C with shaking within 125 mL foil-covered Erlenmeyer flasks under oxic conditions. This was used as the starting culture for inoculating into anoxic media.

### Media Preparation and Sampling

Anoxic media was prepared in 150 mL serum bottles. In total, 50 mL of sterile 100% LB or 10% LB in PBS were amended with various concentrations of sterile NO_3_^–^ or NO_2_^–^ in serum bottles and purged of oxygen. Four nutrient regimes were tested: high carbon and NO_x_^–^ (∼10 mM NO_x_^–^, 100% LB), low carbon (∼10 mM NO_x_^–^, 10% LB), low NO_x_^–^ (∼1 mM NO_x_^–^, 100% LB), and low carbon and NO_x_^–^ (∼1 mM NO_x_^–^, 10% LB). LB concentrations lower than 10% LB did not result in measurable culture growth after 24 h of incubation under the low carbon and NO_x_^–^ regime, therefore 10% LB was chosen to represent the low carbon regime. Within each regime, four initial stoichiometric NO_3_^–^/NO_2_^–^ ratios were tested: 10:0, 9:1, 5:5, and 1:9. Two replicate bottles were prepared for stoichiometric ratios 9:1, 5:5, and 1:9 under each nutrient regime, totaling eight bottles for each along with one abiotic control. For the 10:0 stoichiometric ratio, four replicates were performed, with two sets of bottles sampled on different dates for each nutrient condition. These two sets of bottles were denoted as run 1 and run 2, with the goal to assess reproducibility in growth and denitrification dynamics. Bottles were capped with a butyl rubber stopper and crimped with an aluminum ring to create an airtight seal. Each bottle was purged prior to culture inoculation with N_2_ gas for 2 h at 100 mL min^–1^, resulting in ∼80 volume turnovers. Prepared anoxic bottles were incubated overnight at 37°C without shaking to confirm the sterility of the media prior to inoculation.

Inoculation and sampling were performed with 10 mL syringes which were purged each time prior to insertion into bottles. Purging was performed with N_2_ gas three times as follows: needles were inserted into a capped, sealed empty serum bottle connected to N_2_ gas flowing at 1,000 mL min^–1^. After syringes were filled fully with N_2_ gas, they were removed from the bottle and N_2_ gas was discharged. Holding each syringe stopper down to prevent oxygen from entering the syringe, needles were reinserted into the N_2_ serum bottle and allowed to refill with N_2_ gas. This process ensured that any residual oxygen within each syringe and needle was removed and no oxygen contaminated anaerobic cultures. With the last purge, prior to insertion of the needle into media, 2 mL of N_2_ gas was retained within the syringe and injected into the serum bottle to maintain pressure inside the incubation bottles after sampling.

**FIGURE 1:**
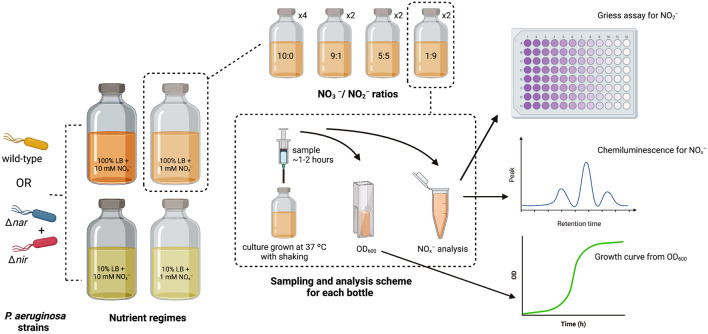
Diagram of experimental setup, sampling scheme, and analysis methods. *P. aeruginosa* strains for inoculation are depicted in orange (wild-type), cyan (Δ*nar*), and red (Δ*nir*). Nutrient regimes are shown in large serum bottles, and stoichiometric ratios for each nutrient regime are shown in smaller serum bottles. Number of replicates for each stoichiometric ratio within each nutrient regime is indicated next to smaller serum bottles. The sampling scheme for each replicate is depicted within the dashed rectangle, along with the analyses performed on each sample.

The optical density (OD) of each overnight aerobic bacterial culture was measured at 600 nm on a Nanodrop One^*C*^ spectrophotometer using a 1 cm tte. For Δ*nir* and Δ*nar* cultures, overnight cultures were combined in a 1:1 cellular ratio within 50 mL Falcon tubes. Either this 1:1 culture mixture or the wild-type culture was added to each bottle to achieve a starting inoculum OD of 0.05. Serum bottles were then placed within a 37°C incubator with shaking. A diagram summarizing the experimental setup, sampling scheme, and analysis methods is included as Figure 1.

Sampling was performed every hour using needles purged as described above. For each sample, 2 mL of media was removed from each bottle. In total, 1 mL of media was preserved in a 1.5 mL microcentrifuge tube for analysis of NO_x_^–^ and NO_2_^–^, while the other 1 mL was measured directly for OD. The tube was centrifuged to pellet cells and the supernatant was transferred to a second tube and frozen at −20°C until analysis for inorganic nitrogen. Bottles were removed from the 37°C incubator only during sampling to minimize time at room temperature, and the total duration of sampling for all bottles at each timepoint was approximately 5 min.

Sampling was terminated when no NO_2_^–^ remained within any cultures. A diagnostic test was performed upon each sample by adding 10 μL of Griess reagent to cuvettes used for OD measurement. If NO_2_^–^ remained, media within cuvettes developed a pink hue, while if NO_2_^–^ was fully consumed, media remained clear. NO_2_^–^ concentrations within preserved tubes were also determined using this method, the Griess colorimetric assay (Strickland and Parsons, 1972). Absorbance was measured on a plate reader at 543 nm using a reference absorption baseline at 750 nm. Total NO_*x*_^–^, or NO_3_^–^ + NO_2_^–^, was determined by chemical reduction to NO with hot acidified vanadium (III) and measured *via* chemiluminescence with a NOx analyzer (Garside, 1982; Braman and Hendrix, 1989). The detection limit for the chemiluminescent NO_x_^–^ method was <0.10 μM. Initial and final pH was taken from a replicate under the same nutrient regimes and culture conditions, with initial pH measured prior to culture inoculation and final pH measured after culture had reached stationary phase.

### Data Analysis

Logistic growth curves were fit to each OD time course and evaluated for goodness of fit. From this analysis, maximum growth rates, saturation points, and lag times were calculated for each replicate in each condition (Zwietering et al., 1990). Growth yields were approximated using fold-change differences between the initial inoculum of each culture and final OD at saturation.

From NO_x_^–^ data, we calculated NO_3_^–^ concentrations as NO_3_^–^ = NO_x_^–^–NO_2_^–^ for each timepoint. Measurements for NO_x_^–^, NO_3_^–^, and NO_2_^–^ were smoothed with a 2nd-degree polynomial Savitsky–Golay filter, which is widely used to filter time series data (Savitzky and Golay, 1964). As NO_x_^–^ measurements, colorimetric NO_2_^–^ concentrations, and calculated NO_3_^–^ include a degree of noisiness, this filter minimizes the influence of noise upon calculated DNRN and denitrification rates. Rates of change for NO_x_^–^, NO_3_^–^, and NO_2_^–^ with time were determined by differentiating with time each curve for each regime, condition, and replicate. DNRN rates were calculated by DNRN = –dNO_3_^–^/dt and denitrification rates were calculated as denitrification = –dNO_x_^–^/dt. Maximum DNRN and denitrification rates were found for each trial. For temporal dynamics for DNRN and denitrification, we delineate three broad categories in our data: synchronous, asynchronous, and contemporaneous. We define the activation of DNRN and denitrification as “synchronous” when the peaks for DNRN and denitrification rates are concurrent, i.e., the second derivatives of concentration with respect to time share the same sign and the maximum rates for DNRN and denitrification occur simultaneously. “Asynchronous” activation is defined as when DNRN rates and denitrification rates do not have maxima at approximately the same time, and rates do not follow the same temporal pattern of change (i.e., the second derivatives of concentration with respect to time have opposite signs). Behaviors in which the curves for DNRN and denitrification rates follow similar upward or downward trends over similar time periods, but do not peak at the same time point are termed “contemporaneous.”

The nitrite accumulation index was defined as NAI = (NO_2_^–^_max_–NO_2_^–^_initial_)/NO_3_^–^_initial_. Analyses were performed in MATLAB release R2018a. We used paired 1-sided and 2-sided *t*-tests as appropriate to evaluate the statistical significance of differences in growth rate, growth yield, nitrite accumulation indices, DNRN rates, and denitrification rates for each nutrient regime for generalists vis-à-vis specialists. The paired *t*-test was used to compare across all stoichiometric ratios of NO_3_^–^/NO_2_^–^ for generalists vs. specialists under each nutrient regime.

## Results

To confirm that the *Pseudomonas aeruginosa* PAO1 Δ*nar* mutant could respire NO_2_^–^ but not NO_3_^–^, the Δ*nir* mutant could respire NO_3_^–^ but not NO_2_^–^, and the wild-type (WT) strain could respire both, we grew all strains axenically under anoxic conditions for 27 h in LB media supplemented with 10 mM of NO_3_^–^ or NO_2_^–^. As anticipated, the Δ*nar* mutant could only grow under 10 mM NO_2_^–^ but not under NO_3_^–^ (Supplementary Figure 1A). The Δ*nir* mutant could not grow under NO_2_^–^, but could grow under NO_3_^–^. WT grew under both conditions, indicating that 10 mM NO_2_^–^ did not inhibit its growth, and grew better given NO_3_^–^ as it could harness the additional energy of the first denitrification step. In addition, the Δ*nar* mutant reached the same optical density (OD) as the wild-type under 10 mM NO_2_^–^, indicating that 10 mM NO_2_^–^ did not inhibit its growth either. These results show that the Δ*nar* mutant did indeed lose the function of the *nar* gene responsible for nitrate reductase but maintained the remainder of the denitrification pathway. Likewise, the Δ*nir* mutant lost the function of the *nir* gene responsible for nitrite reductase but retained the function of *nar* and likely *nor* and *nos*. As all mutants reached an OD of ∼0.5 or higher within 27 h, and lag times for co-cultures (Supplementary Figure 2) approach those of axenic wild-type in several nutrient conditions, we do not expect growth or regulatory defects from these gene deletions to substantially impact our results.

To test whether the Δ*nar* and Δ*nir* mutants performed substrate cross-feeding in co-culture, we compared the growth of axenic Δ*nar* and Δ*nir* cultures against a co-culture of Δ*nar* and Δ*nir* (Δ + Δ) under anoxic conditions in LB supplemented with 1, 10, or 100 mM NO_3_^–^. We found the Δ + Δ co-culture grew under all initial NO_3_^–^ conditions, while the axenic Δ*nar* did not grow (Supplementary Figure 1B). The final co-culture OD surpassed the final OD of the axenic Δ*nir* strain under all conditions, indicating that growth was not simply due to the Δ*nir* strain within the co-culture but that the two strains performed metabolite cross-feeding. The Δ + Δ co-culture surpassed the growth of the axenic Δ*nir* strain under 1 mM NO_3_^–^, so it is unlikely that this result was due to the toxicity of accumulated NO_2_^–^ within the media. In addition, the axenic Δ*nir* reached a higher OD under 10 mM NO_3_^–^ and 100 mM NO_3_^–^ than under 1 mM NO_3_^–^, indicating its ability to tolerate higher NO_2_^–^ concentrations.

**FIGURE 2:**
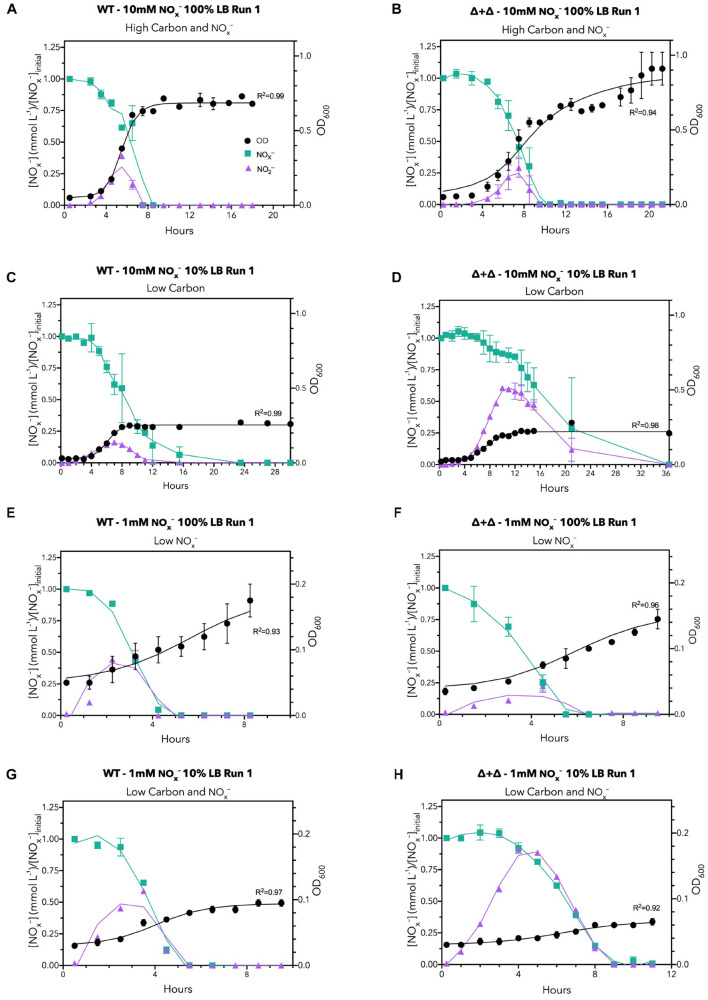
Time series data under all LB nutrient regimes for 10:0 NO_3_^–^/NO_2_^–^ ratios for the first run. The regimes are: **(A,B)** high carbon and NO_x_^–^ **(C,D)** low carbon, **(E,F)** low NO_x_^–^ and **(G,H)** low carbon, and NO_x_^–^. NO_x_^–^ (green), NO_2_^–^ (purple), and bacterial growth (black) are shown. Left hand panels correspond to the wild-type culture whereas right hand panels depict the mutant co-culture. NO_x_^–^ data are normalized to the initial NO_x_^–^ concentration during plotting to reduce variability introduced by chemiluminescent measurements and variations in initial NO_x_^–^ loading. Data points are the means of two biological replicates per condition, with error bars indicating ranges. Curves for NO_x_^–^ and NO_2_^–^ were smoothed with a Savitsky-Golay filter, while OD_600_ curves were fit to a logistic growth model. Selected plots are presented for brevity; analogous plots for other replicates and initial NO_3_^–^/NO_2_^–^ ratios can be found in Supplementary Figures 6–9.

The dynamics of NO_x_^–^ consumption, NO_2_^–^ accumulation, and growth over the time course of sampling for each nutrient regime for two replicates for the 10:0 NO_3_^–^/NO_2_^–^ ratio is displayed in Figure 2. Under a high carbon and NO_x_^–^ regime (10 mM NO_x_^–^ and 100% LB), the OD of all cultures reached greater than 0.5, but when available carbon was reduced by a factor of 10, growth decreased to an OD of 0.2–0.4 (Figure 2), indicating that growth was reduced by the lower amount of carbon. When carbon availability was kept high with 100% LB but available nitrogen was decreased to 1 mM NO_x_^–^, growth decreased to an OD of 0.15–0.25 (Figure 2), demonstrating growth limitation by NO_x_^–^. When both carbon and nitrogen availability were low in the low carbon and NO_x_^–^ regime (1 mM NO_x_^–^ and 10% LB), the final OD was further depressed compared to both low carbon and low NO_x_^–^ regimes, to an OD of approximately 0.1 (Figure 2), indicating growth was depressed by starvation for carbon and NO_x_^–^. The control experiments in M9 minimal media showed no decrease in OD between 50 mM citrate (300 mM carbon) and 5 mM citrate (30 mM carbon) for the same NO_3_^–^ concentrations (Supplementary Figure 3). This may be due to specifics of carbon metabolic processing or the total bioavailability of labile carbon derived from cellular material compared to citrate (Rojo, 2010; Dolan et al., 2020).

**FIGURE 3:**
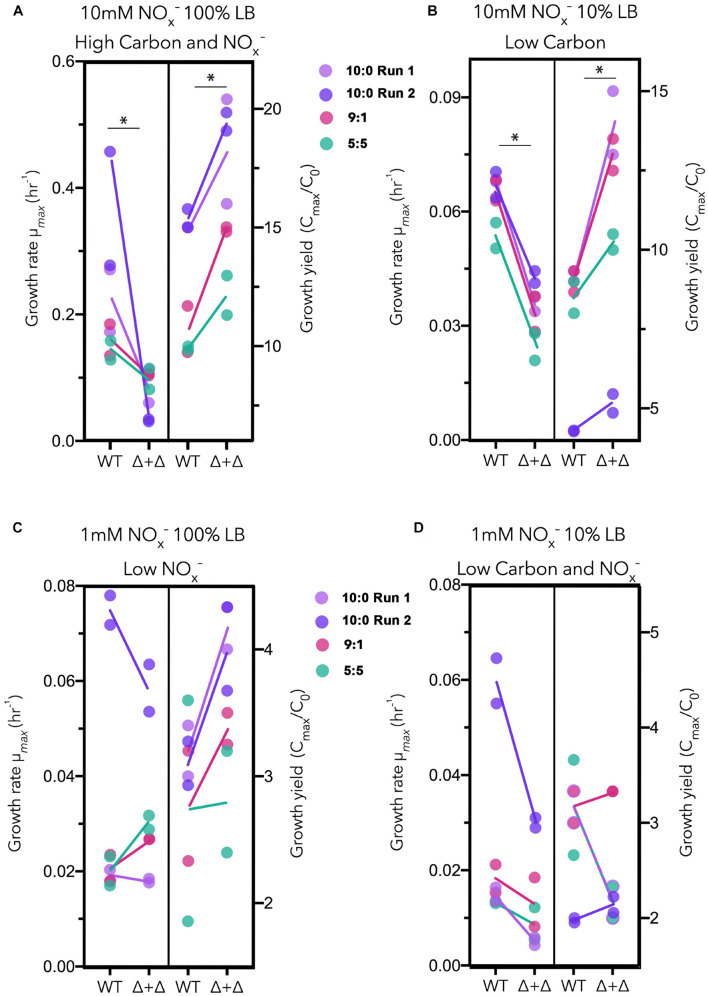
Growth rate vs. growth yield for generalists and specialists in LB. Results for three stoichiometric NO_3_^–^/NO_2_^–^ ratios (10:0 run 1, lavender; 10:0 run 2, dark purple; 9:1, red; 5:5, green) are plotted together for each nutrient regime as follows: **(A)** high carbon and NO_x_^–^, **(B)** low carbon, **(C)** low NO_x_^–^, and **(D)** low carbon and NO_x_^–^. Maximum growth rates (μ_max_) vs. growth yields (C_max_/C_0_) are plotted separately for wild-type (WT) and Δ*nar* + Δ*nir* co-culture (Δ + Δ). For both WT and Δ + Δ, *n* = 4 for the 10:0 NO_3_^–^/NO_2_^–^ ratio and *n* = 2 for the 9:1 and 5:5 ratio for each nutrient regime, and *n* = 8 for each nutrient regime including all depicted ratios, omitting 1:9.

For each culture, we calculated the maximum growth rate and the approximate growth yield, represented in Figure 3. We approximate growth yields by taking the fold-change between the cell density of the starting culture and the maximum cell density based on OD. Under high carbon and NO_x_^–^ and low carbon regimes, in which nitrogen was high, Δ + Δ co-cultures achieved a higher growth yield (high carbon and NO_x_^–^: *n* = 8, paired 1-sided *t*-test, *p* = 0.0002; low carbon: *n* = 8, paired 1-sided *t*-test, *p* = 0.002), while WT exhibited a higher maximum growth rate (high carbon and NO_x_^–^: *n* = 8, paired 1-sided *t*-test, *p* = 0.001; low carbon: *n* = 8, paired 1-sided *t*-test, *p* = 6.5 × 10^–7^) (Figure 3). However, when NO_x_^–^ was low at 1 mM, the relationship between growth rate and growth yield for the Δ + Δ co-cultures compared to WT changed. Under low NO_x_^–^ regimes, the differences between growth rate and growth yield for each culture were non-significant (*p* > 0.01 as determined by a 1-sided *t*-test). Under low carbon and NO_x_^–^ regimes, WT had higher maximum growth rates (*n* = 8, paired 1-sided *t*-test, *p* = 0.009) but growth yields were not significantly different (*n* = 8, paired 1-sided *t*-test, *p* = 0.08). For these statistical tests, we used 10:0, 9:1, and 5:5 NO_3_^–^/NO_2_^–^ stoichiometric ratios and excluded the 1:9 stoichiometric ratio as the low NO_3_^–^ availability could not support substantial growth of the obligate NO_2_^–^ producer within the co-culture. Growth effects for the 1:9 ratio more likely result from the lower initial inoculum sizes of cells capable of utilizing NO_2_^–^ in the Δ + Δ co-cultures compared to the WT, and little potential for cross-feeding exists between the two mutants, particularly in the 1 mM NO_x_^–^ regimes. Previous studies indicate the precise context in terms of the type of carbon compound is key (Rojo, 2010; Dolan et al., 2020), and our control experiments with a single carbon source also suggest a possible growth yield vs. growth rate tradeoff between WT and Δ + Δ under all nutrient regimes, warranting follow-up study to investigate species specific responses to carbon affinity (Supplementary Figure 4A).

**FIGURE 4:**
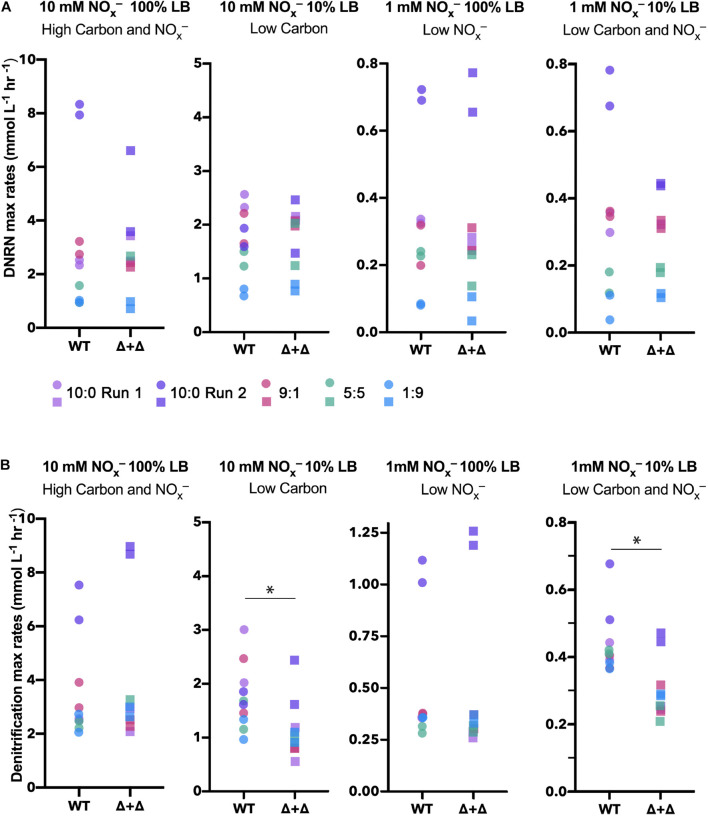
Maximum DNRN and denitrification rates for WT and Δ + Δ cultures in LB. Maximum DNRN rates are plotted for each culture under each nutrient condition and colored by NO_3_^–^/NO_2_^–^ ratio (10:0 run 1, lavender; 10:0 run 2, dark purple; 9:1, red; 5:5, green; 1:9, blue). For both WT and Δ + Δ, *n* = 4 for the 10:0 NO_3_^–^/NO_2_^–^ ratio and *n* = 2 for the 9:1 and 5:5 ratio for each nutrient regime, and *n* = 10 for each nutrient regime including all depicted ratios. There is no significant difference in DNRN rates when comparing between WT or Δ + Δ for any treatment. DNRN rates are 10-fold higher under 10-fold higher NO_x_^–^, but are not influenced by carbon availability. **(B)** Maximum denitrification rates for cultures and conditions, as in panel **(A)**. For 10% LB regimes, denitrification rates decrease in Δ + Δ cultures, while there is no difference between denitrification rates when comparing cultures for 100% LB regimes. NO_3_^–^/NO_2_^–^ ratios did not significantly affect denitrification rates.

From total nitrogen oxyanion (NO_x_^–^) concentrations measured for each time point, we found a loss of NO_x_^–^ from both the WT and Δ + Δ co-cultures (Figure 2). For the Δ + Δ co-cultures, this demonstrates that the obligate NO_2_^–^ producer (Δ*nir*) reduced NO_3_^–^ to NO_2_^–^, and the obligate NO_2_^–^ consumer (Δ*nar*) further reduced NO_2_^–^. Distinct temporal dynamics of nitrogen oxyanion (NO_x_^–^) consumption distinguish Δ + Δ co-cultures and axenic WT. In addition to measuring total NO_x_^–^ over time, we also measured NO_2_^–^ over time and, from the curves of NO_x_^–^ and NO_2_^–^, calculated NO_3_^–^ over time. Assuming that both mutants could respire NO and N_2_O, we focused our analysis on the reduction of NO_3_^–^ to NO_2_^–^ and the loss of NO_2_^–^, which we respectively differentiate as DNRN and denitrification. Although DNRN canonically represents the initial reaction of the denitrification pathway, fixed nitrogen is not lost as the resulting NO_2_^–^ remains bioavailable. However, the reduction of NO_2_^–^ to gaseous forms of nitrogen results in the loss of bioavailable nitrogen from the system, and this step is considered the defining reaction of denitrification. We found the rates of DNRN and denitrification in all conditions, as shown in Figure 4. DNRN rates did not vary between WT or co-culture in any condition (*n* = 40, paired 2-sided *t*-test, *p* = 0.3) (Figure 4A). Notably, DNRN rates were 10-fold higher in the 10 mM NO_x_^–^ regimes compared to the 1 mM NO_x_^–^ regimes. At higher initial NO_3_^–^ conditions (10:0, 9:1), DNRN rates were highest, whereas DNRN rates were lowest under the 1:9 NO_3_^–^/NO_2_^–^, condition. This reveals that the major determinant of DNRN rate is the amount of NO_3_^–^ available and that both WT and the Δ + Δ co-culture reduce NO_3_^–^ with equal speed. In contrast, denitrification rates were statistically indistinguishable between cultures growing in 100% LB (*n* = 20, paired 2-sided *t*-test, *p* = 0.2) but denitrification rates were lower for Δ + Δ co-cultures compared to wild-type in 10% LB (*n* = 20, paired 1-sided *t*-test, *p* = 0.006) (Figure 4B). This difference was observed for all stoichiometric NO_3_^–^/NO_2_^–^ ratios in LB.

**FIGURE 5:**
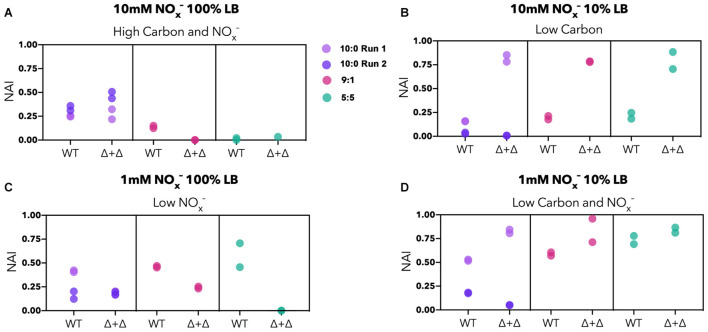
NO_2_^–^ accumulation index (NAI) for generalist and specialist cultures in LB. Stoichiometric ratios are plotted separately for Δ + Δ co-cultures and WT for each nutrient regime (10:0 run 1, lavender; 10:0 run 2, dark purple; 9:1, red; 5:5, green). For both WT and Δ + Δ, *n* = 4 for the 10:0 NO_3_^–^/NO_2_^–^ ratio and *n* = 2 for the 9:1 and 5:5 ratio for each nutrient regime, and *n* = 8 for each nutrient regime including all depicted ratios, omitting 1:9. NAI relates to synchronicity, with synchronous cultures exhibiting low NAI and asynchronous cultures exhibiting high NAI. **(A)** The NAI is low to moderate for high carbon and NO_x_^–^ regimes; **(B)** NAI is generally higher for Δ + Δ for low carbon regimes, but variable between replicates; **(C)** NAI is generally higher for WT for low NO_x_^–^ regimes; **(D)** NAI can reach high values for low carbon and NO_x_^–^ but is variable between replicates.

To examine whether the NO_2_^–^ accumulation differs between specialists and generalists, we compared the NO_2_^–^ accumulation index (NAI) for each nutrient condition. NAI = 1 indicates all NO_3_^–^ was reduced quantitatively to NO_2_^–^ before NO_2_^–^ reduction commenced whereas NAI = 0 reflects no transient NO_2_^–^ accumulation. For the high carbon and NO_x_^–^ regime, NO_2_^–^ accumulates to a moderate extent in the 10:0 ratio (NAI = 0–0.5), and Δ + Δ co-cultures and WT do not differ significantly from each other (*n* = 8, paired 2-sided *t*-test, *p* = 0.9). The highest NAI values occurred in the Δ + Δ co-cultures under low carbon, reaching almost 100% of the initial nitrogen loading, significantly higher than WT under the same conditions (*n* = 8, paired 1-sided *t*-test, *p* = 0.002) (Figure 5). For the low NO_x_^–^, high carbon regime, WT cultures generally reached higher NAI than Δ + Δ co-cultures (*n* = 8, paired 1-sided *t*-test, *p* = 0.01). In the single carbon control, WT cultures did not accumulate measurable NO_2_^–^ in any condition whereas Δ + Δ co-cultures consistently accumulated this intermediate (Supplementary Figure 4B). However, high variability was observed in NAI between replicates started from different inocula for the 10:0 ratio, indicating additional controls on NAI beyond nutrient condition or metabolic lifestyle.

In addition to maximum rates and NO_2_^–^ changes, we explored the temporal dynamics of DNRN and denitrification during growth. We did not find clear partitioning of synchronicity according to either nutrient regime or specialist vs. generalist cultures (Supplementary Figure 5). The observed patterns of synchronicity follow the extent of NO_2_^–^ accumulation. For cultures in LB exhibiting asynchronous DNRN and denitrification dynamics, more NO_2_^–^ accumulates. Conversely, cultures exhibiting synchronous DNRN and denitrification accumulate less NO_2_^–^, and contemporaneous cultures accumulate some NO_2_^–^ but not to levels as high as asynchronous cultures. In all conditions, DNRN always proceeds prior to denitrification. These patterns suggest that synchronicity, rather than maximal DNRN or denitrification rates, drives the accumulation of NO_2_^–^.

In general, Δ + Δ co-cultures required a longer time to begin growth, perform DNRN and denitrification, and reach the stationary phase. Lag times, defined as the period prior to cell division and exponential growth, are generally higher for Δ + Δ co-cultures vs. the WT PAO1 across all regimes (Supplementary Figure 2). The onset of DNRN and denitrification in Δ + Δ was generally slower than in WT, possibly reflecting the lower initial density of denitrification-capable cells (Δ*nar*). The duration of time from inoculation to the total consumption of NO_x_^–^ was consistently longer in Δ + Δ than in WT (Supplementary Figures 6–9), reflective of the same growth rate v. yield tradeoff between WT and Δ + Δ cultures. OD curves indicate that the Δ + Δ co-cultures require longer to reach saturation, which is consistent with the observation that logarithmic growth corresponds with the period of DNRN and denitrification activity.

## Discussion

The results from this study, examining a high carbon and NO_x_^–^ regime for the axenic *Pseudomonas aeruginosa* PAO1 wild-type (WT) generalist compared to co-cultured DNRN and denitrification (Δ + Δ) specialists, are consistent with a growth rate vs. growth yield tradeoff. Our results in both LB and M9 media offer evidence for this tradeoff. Growth rate and growth yield, a proxy for net growth efficiency, are two fundamental traits of microbes that influence community function, evolution, and species coexistence (Lipson, 2015). This rate vs. yield tradeoff has been found within laboratory evolution experiments using diverse organisms such as *E. coli* (Novak et al., 2006), *Lactobacillus lactis* (Bachmann et al., 2013), and yeasts (Weusthuis et al., 1994), as well as in natural microbial communities (Sorokin et al., 2003; Lipson et al., 2009). However, to our knowledge, this tradeoff has not previously been experimentally demonstrated using model generalists and specialists within the same strain in the context of varying nutrient regimes.

Previous numerical approaches have shown that the rate vs. yield tradeoff is not universal, but depends upon environmental conditions (Lipson et al., 2009; Beardmore et al., 2011). We find this tradeoff to be consistent for nutrient replete and low carbon conditions. When the amount of NO_x_^–^ (electron acceptor) is decreased, some Δ + Δ co-cultures reach higher growth rates compared to WT and some WT cultures reach higher growth yields than Δ + Δ co-cultures (Figure 3). Metabolic savings alone are unlikely to explain this, so it may be useful to interpret this result through the lens of ecological interactions within each culture. Within axenic WT, each cell competes with others for NO_x_^–^ and carbon. However, within a Δ + Δ co-culture, competitive pressure for NO_x_^–^ is reduced as only a portion of the population can use NO_3_^–^ while the other portion can only use NO_2_^–^. In addition, obligate NO_2_^–^ consumers engage in a commensal relationship with obligate NO_2_^–^ producers. Previous studies have demonstrated the differential effects of competition or commensalism on the spatial arrangements and communal behaviors of interacting denitrifiers (Hibbing et al., 2010; Faust and Raes, 2012; Lilja and Johnson, 2019; Ciccarese et al., 2020). Under a low NO_x_^–^ regime, competition for NO_x_^–^ may complicate the terms of the rate vs. yield tradeoff. Additionally, in nutrient-depleted conditions, low cell density may impede efficient substrate exchange between separate specialist populations. Further experiments are required to pinpoint the conditions under which the rate vs. yield tradeoff changes and the mechanisms, ecological or physiological, underlying this change.

The presence of metabolic division of labor generally correlates with increased potential accumulation of NO_2_^–^ and other metabolic intermediates in natural environments. However, accumulation of NO_2_^–^ may also occur in complete denitrifiers under certain conditions, and displays high variability even for cultures with the same genetic content growing under the same nutrient conditions. The accumulation of NO_2_^–^ also changes for cultures growing in mixed vs. single carbon sources, as complexity in carbon resources will likely modify thermodynamic and kinetic stimuli (Rojo, 2010). Over the course of the cultures, interactions in the co-culture may lead to varied growth dynamics of each mutant. These specific dynamics, while not captured in our experimental scheme, may clarify this variability between cultures and serves as a basis for future experimental work. Additionally, further studies on the regulation of the denitrification pathway, the link between denitrification and carbon metabolism in generalist and specialist species, and the individual growth dynamics of each specialist within co-cultures are required to determine the exact drivers of intermediate accumulation and its impacts on denitrifying community behavior.

The accumulation of NO_2_^–^ does not track with differences in maximal DNRN or denitrification rates between cultures or nutrient conditions. Obligate NO_2_^–^ producers do not exhibit lower rates of NO_3_^–^ conversion compared to wild-type generalists under any nutrient condition (Figure 4A). In contrast, obligate NO_2_^–^ consumers maintain decreased rates of NO_2_^–^ reduction compared to generalists only when carbon is low (Figure 4B). This indicates different sensitivities or regulations of *nar* and *nir* toward nutrient availability and type. However, differential accumulation of NO_2_^–^ between WT and co-cultures occurred in low NO_x_^–^ conditions, which displayed little difference in DNRN or denitrification rates. This may be explained by the temporal dynamics of DNRN and denitrification. Cultures exhibiting low NAI are more synchronous than cultures exhibiting high NAI (Supplementary Figure 5). For the WT, this synchronicity points to simultaneous activation of both portions of the denitrification pathway, but for the co-culture synchronous DNRN and denitrification indicates simultaneous metabolism by both specialists. Asynchronous behavior in the WT reveals a temporal delay between DNRN and denitrification, possibly due to regulatory differences in gene expression between *nar* and *nir* (Körner and Zumft, 1989; Schreiber et al., 2007). In the Δ + Δ co-culture, asynchrony indicates population succession, with the obligate NO_2_^–^ producer growing first followed later by the obligate NO_2_^–^ consumer. The only conditions under which specialists exhibit generally more synchronous behavior than WT are low NO_x_^–^. The ability of both populations to grow non-exclusively points to a commensal, rather than competitive, interaction for a scarce nutrient. This commensal interaction correlates with the only regime (low NO_x_^–^) in which NAI is lower for the Δ + Δ co-culture than for the WT (Figure 5). Broadly, these results suggest that the temporal dynamics rather than the maximal rates of the individual steps of denitrification may drive the extent of intermediate accumulation. Further work on individual dynamics of growth for each mutant and timing of *nar* and *nir* transcription may shed light upon the mechanisms underlying these behaviors.

*Pseudomonas aeruginosa*, along with many other denitrifying and non-denitrifying organisms, possesses an additional nitrate reductase system, periplasmic nitrate reductase enzyme Nap encoded by the *nap* gene (Alst et al., 2009). As *nap* was not deleted in our system, this may potentially influence NO_3_^–^ reduction. However, since Nap cannot generate a proton motive force for ATP synthesis and growth, it is unlikely that Nap played a large role in the growth dynamics observed. Additionally, *nap*, along with fermentative processes, has been shown to activate mostly in the stationary phase, while *nar* is expressed during active growth (Alst et al., 2009; Schiessl et al., 2019). As the dynamics we observe are based upon pre-stationary phase growth and metabolism under anoxic conditions, we do not expect either of these processes to be a substantial influence. However, the regulation of the denitrification pathway is complex, so the influence of *nap* cannot be ruled out and requires further investigation. The role of *nap* in denitrification dynamics, denitrifier evolution, and metabolic niche differentiation is an exciting complementary research avenue.

Using engineered strains of *Pseudomonas aeruginosa* PAO1, we compare the behavior of complete denitrifiers against a community in which the denitrification pathway has been partitioned between obligate NO_2_^–^ producers and consumers. Our results indicate a growth rate vs. growth yield tradeoff between complete denitrifiers, or generalists, and partial denitrifiers, or specialists under nutrient replete and high NO_x_^–^ conditions. While few studies have surveyed complete vs. partial denitrifiers across various environments, several studies on denitrifying communities reveal a high prevalence of partial denitrifiers in soils and wetlands (Roco et al., 2017). A study of metagenome-assembled genomes from various environments also discovered a higher ratio of complete:partial denitrifiers in built environments and in marine and brackish systems (Hester et al., 2019). Relatively richer nutrient conditions and spatial segregation in soils and wetlands may select for metabolic specialization, while more nutrient-limited environments may select for complete denitrifiers. However, more work is required to link the prevalence of complete vs. partial denitrifiers across environments and their nutrient contexts.

We find that nutrient availability, relative amounts of carbon to NO_x_^–^, and the composition of metabolic lifestyles within a denitrifying system play key roles in driving the rate vs. yield tradeoff, the dynamics of NO_x_^–^ consumption, and the accumulation of chemical intermediates. Our data provide evidence of the differences in the growth and denitrification behavior between a community of specialists and generalists, but variability between replicates in relation to the extent of NO_2_^–^ accumulation indicates a complexity in the denitrification pathway that remains to be resolved. Denitrification regulation, bacterial carbon and nitrogen metabolism, the specific thermodynamics driving complete vs. partial denitrification, and the ecological and chemical interactions among denitrifying microbes are likely to be fruitful avenues of future investigation.

## Data Availability Statement

The original contributions presented in the study are included in the article/Supplementary Material, further inquiries can be directed to the corresponding author/s.

## Author Contributions

IZ, SM, DC, SS, and AB analyzed the data and wrote the manuscript. SM conducted the experiments. IZ, SM, DD, and DM analyzed nitrogen samples. MT and NN provided knockout strains for this research. AB designed the study and supervised the project. All authors contributed to the article and approved the submitted version.

## Conflict of Interest

The authors declare that the research was conducted in the absence of any commercial or financial relationships that could be construed as a potential conflict of interest.

## Publisher’s Note

All claims expressed in this article are solely those of the authors and do not necessarily represent those of their affiliated organizations, or those of the publisher, the editors and the reviewers. Any product that may be evaluated in this article, or claim that may be made by its manufacturer, is not guaranteed or endorsed by the publisher.
