# Supplementary material for: Ratio of Electron Donor to Acceptor Influences Metabolic Specialization and Denitrification Dynamics in Pseudomonas aeruginosa in a Mixed Carbon Medium
Source: Front Microbiol. 2021 Sep 10;12:711073. doi: 10.3389/fmicb.2021.711073 (PMC8461185; doi:10.3389/fmicb.2021.711073)
Supplement: Supplementary file 1 [file Data_Sheet_1.docx]

Supplementary Material

**Ratio of electron donor to acceptor influences metabolic specialization
and denitrification dynamics in *Pseudomonas aeruginosa* in a mixed carbon medium**

Irene H Zhang, Susan Mullen, Davide Ciccarese, Diana Dumit, Donald E Martocello III,
Masanori Toyofuku, Nobuhiko Nomura, Steven Smriga, Andrew R Babbin

The supplementary materials include:

**Supplementary Table 1. Primers for the deletion of *narG* and *nirS-N* in *P. aeruginosa* PAO1.**

**Supplementary Figure 1. Endpoint growth response comparison of strains.**

**Supplementary Figure 2. Lag times for cultures in LB across nutrient regimes.**

**Supplementary Figure 3. Time series data under all nutrient regimes in M9 minimal media supplemented with NO_3_^–^ and citrate.**

**Supplementary Figure 4. Growth rates vs. growth yields and NO_2_^–^ accumulation index (NAI) for all nutrient regimes in M9 minimal media.**

**Supplementary Figure 5. Synchronicity of DNRN and denitrification for cultures in LB across nutrient regimes.**

**Supplementary Figure 6.** **Time series data under 10 mM NO_x_^–^ and 100% LB conditions (high carbon and NO_x_^–^).**

**Supplementary Figure 7.** **Time series data under 10 mM NO_x_^–^ and 10% LB conditions (low carbon).**

**Supplementary Figure 8.** **Time series data under 1 mM NO_x_^–^ and 100% LB conditions (low NO_x_^–^).**

**Supplementary Figure 9.** **Time series data under 1 mM NO_x_^–^ and 10% LB conditions (low carbon and NO_x_^–^).**

| **Primers** | **Sequence** | **Source or Reference** |
| --- | --- | --- |
| Δ*nirS*-NF1 | 5′-CGGAATTCCCAGTAGAGGGTGAAGAAAGTGTCGTGC-3′ | Toyofuku et al. (2014) |
| Δ*nirS*-NR1 | 5′-CGGGATCCGACCGGAATCAAGATTGCGTTGCTTTGC-3′ | Toyofuku et al. (2014) |
| Δ*nirS*-NF2 | 5′-CGGGATCCGGTCGAGACGCCATCAACGGATATCC-3′ | Toyofuku et al. (2014) |
| Δ*nirS*-NR2 | 5′-CCGAAGCTTGCGATGTTCACCATGATGAACTACGAGC-3′ | Toyofuku et al. (2014) |
| Δ*narG-*F: | 5′-GGGGATCCCGGCAACCTCTGATTAGCGTTGTAACGC-3′ | This study |
| Δ*narG-*R1: | 5′-CACTAGTGCGCACCTCGGTGAAGAAGTGCG-3′ | This study |
| Δ*narG-*R: | 5′-GGGAAGCTTGGCGTCTCTCCTCAAATGTCCTGGG-3′ | This study |
| Δ*narG-*F2: | 5′-CACTAGTCTGGGAAGCGCTGTCGAAGATCACC-3′ | This study |

**Supplementary Table 1. Primers for the deletion of *narG* and *nirS-N* in *P. aeruginosa* PAO1.** Oligonucleotide primers, primer sequences, and references if applicable are listed for each primer used to generate deletion mutants.

**
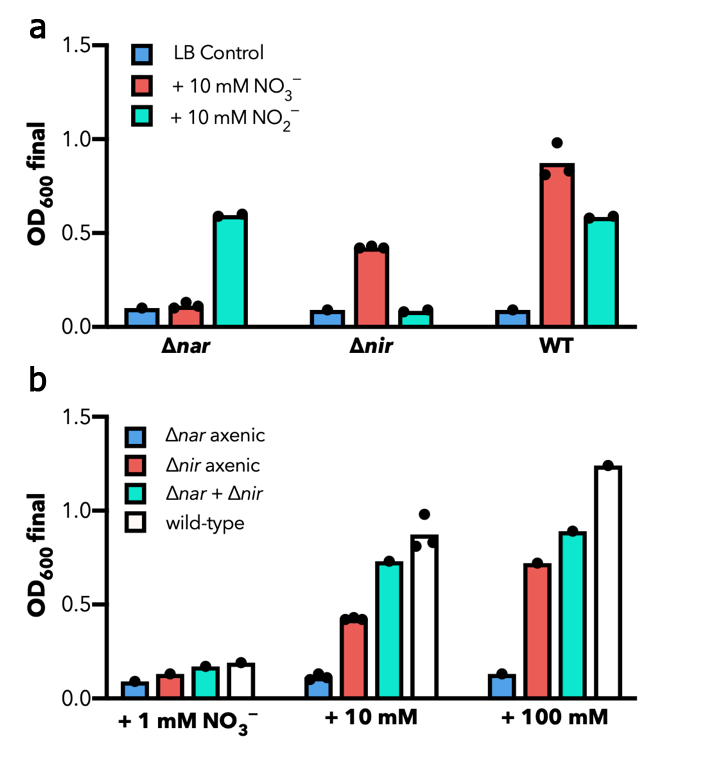
**

**Supplementary Figure 1. Endpoint growth response comparison of strains.** **a** Axenic strains were grown in anoxic LB media (control) or LB supplemented with 10 mM NO_3_^–^ or NO_2_^–^. Optical density was measured after 27 hours. Dots represent replicate values for each treatment. Mutants responded as expected, specifically, Δ*nar* respired NO_2_^–^ but not NO_3_^–^, Δ*nir* respired NO_3_^–^ but not NO_2_^–^, and WT respired both. Media was inoculated with each strain to an initial density of OD_600_ = 0.1. **b** Axenic strains were grown in anoxic LB media (control) or LB supplemented with 1, 10, or 100 mM NO_3_^–^. Optical density was measured after 24 hours. Dots represent replicate values for each treatment.


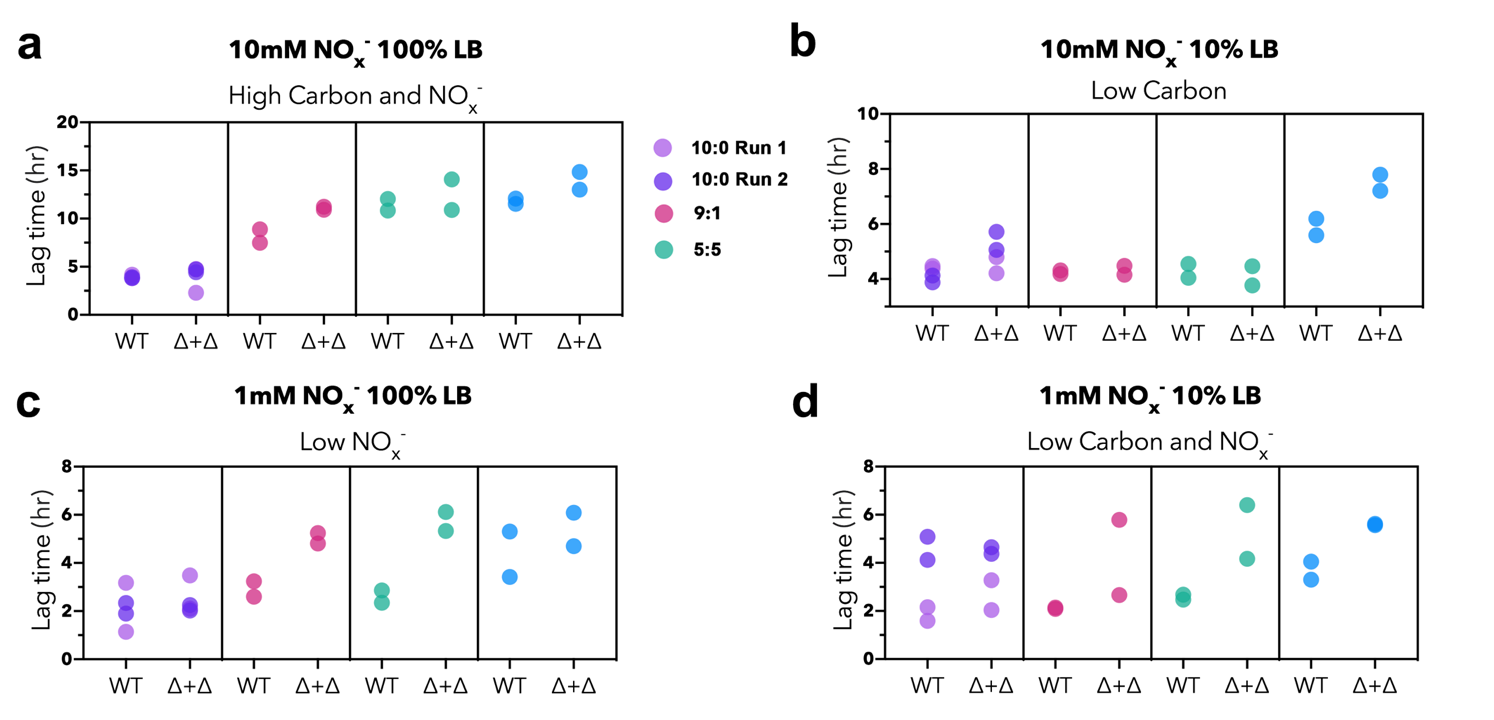


**Supplementary Figure 2: Lag times for cultures in LB across nutrient regimes.** Lag times are plotted by WT vs. Δ+Δ cultures for each stoichiometric NO_3_^–^/NO_2_^–^ ratio (10:0 Run 1, lavender; 10:0 Run 2, dark purple, 9:1, pink; 5:5, green; 1:9, blue) as follows: **a** high carbon and NO_x_^–^ **b** low carbon **c** low NO_x_^–^ and **d** low carbon and NO_x_^–^. Lag time, or the period prior to the logarithmic phase of bacterial growth as measured by OD_600_, is generally longer for Δ+Δ cultures except under 10:0 ratios.­

**Supplementary Figure 3. Time series data under all nutrient regimes in M9 minimal media supplemented with a, b** 10 mM NO_3_^–^ and 50 mM citrate **c** 10 mM NO_3_^–^ and 5 mM citrate **d, e** 1 mM NO_3_^–^ and 50 mM citrate **f, g** 1 mM NO_3_^–^ and 5 mM citrate. NO_x_^–^ concentrations (green), NO_2_^–^ (purple), and bacterial growth (black) are shown. Curves for NO_x_^–^ and NO_2_^–^ were smoothed with a Savitsky-Golay filter, while OD_600_ curves were fit to a logistic growth model. Wild-type *P. aeruginosa* data are missing for the low carbon (10mM NO_3_^–^ 30 mM C) regime due to contamination.


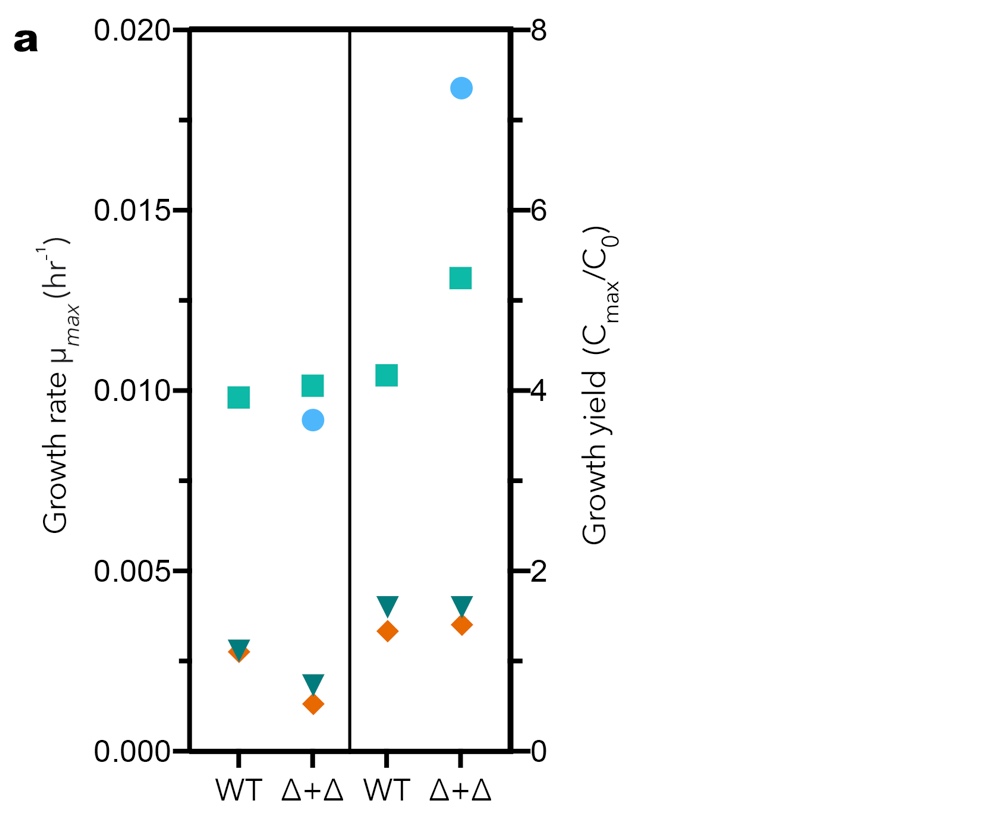


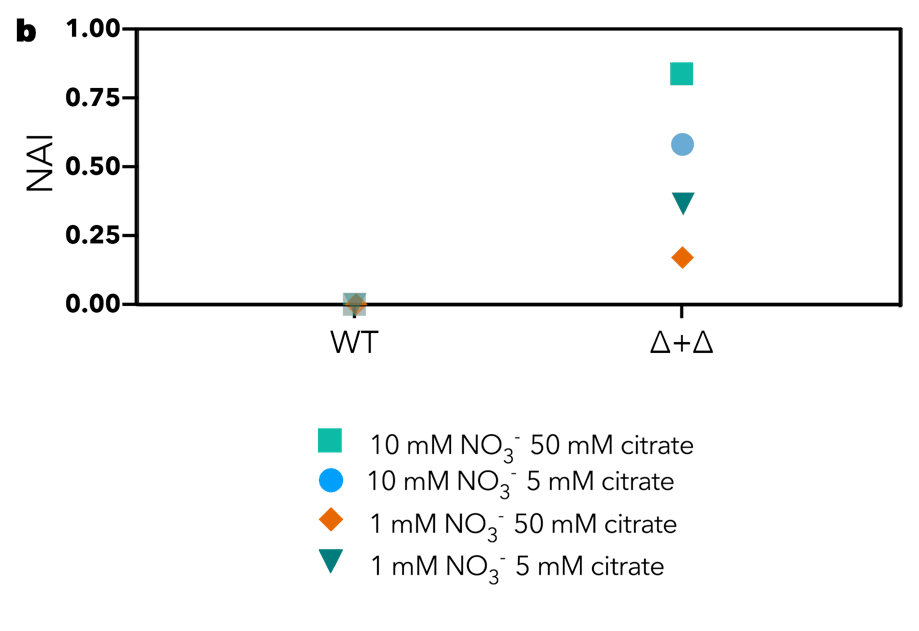


**Supplementary Figure 4. Wild-type (WT) vs. Δ*nir* +Δ*nar* co-cultures (Δ+Δ) grown in M9 minimal media supplemented with NO_3_^–^ and citrate showing a** maximum growth rates (µ_max_) vs. growth yields (C_max_/C_0_) **b** NO_2_^–^ accumulation index (NAI). Results for all nutrient regimes are plotted together for brevity and colored by regime (teal square: 10 mM NO_3_^–^ 50 mM citrate, blue circle: 10 mM NO_3_^–^ 5 mM citrate, orange diamond: 1 mM NO_3_^–^ 50 mM citrate, green triangle: 1 mM NO_3_^–^ 5 mM citrate). Points represent a single trial.


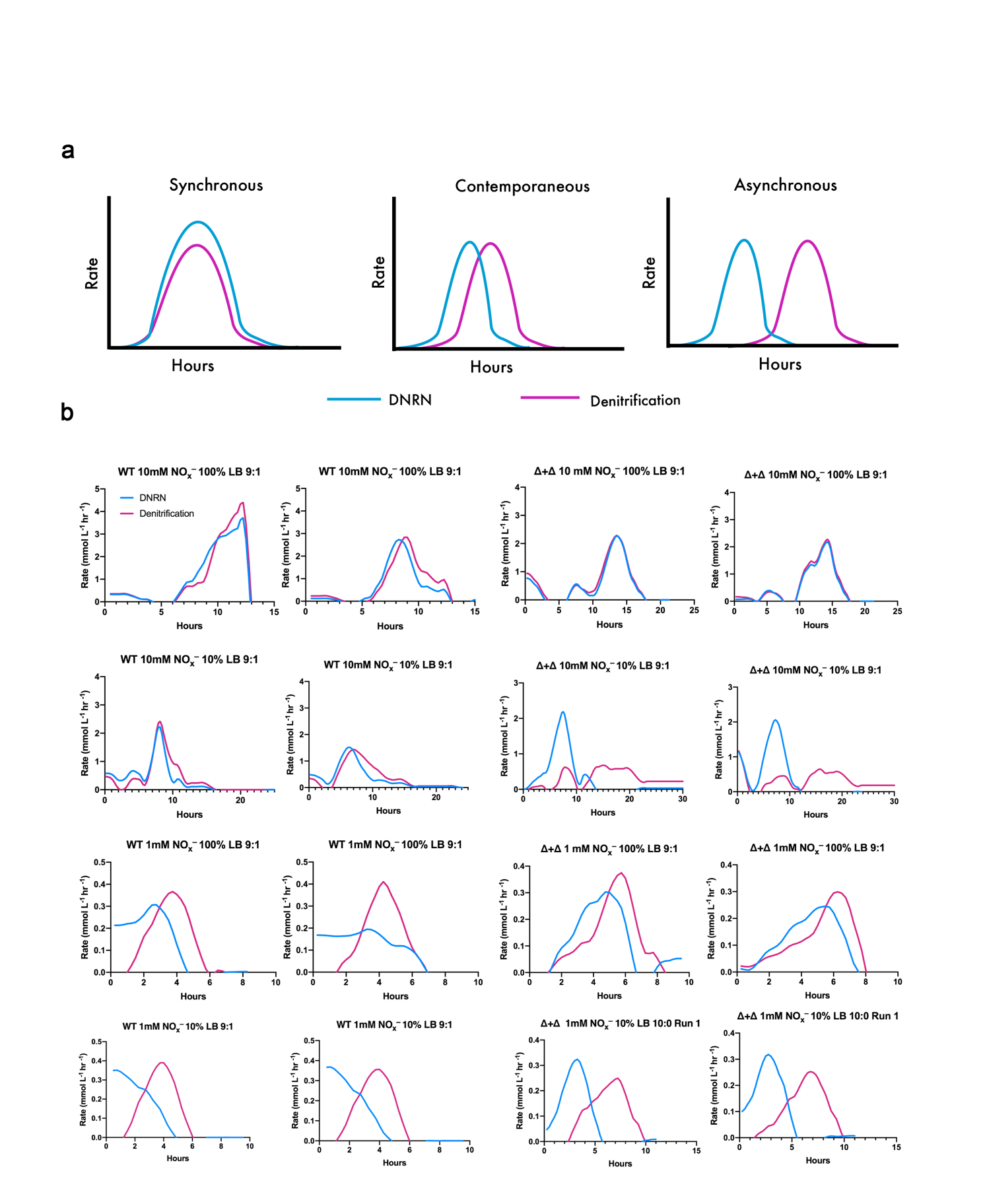


**Supplementary Figure 5: Synchronicity of DNRN and denitrification for cultures in LB across nutrient regimes. a**, idealized representations of three overall categories of DNRN (blue) and denitrification (pink) temporal dynamics: synchronous, contemporaneous, and asynchronous. **b**, plots from each condition and run plotted over time for the WT and Δ+Δ cultures under each nutrient regime as follows: row 1, 10 mM NO_x_^–^ 100% LB; row 2, 10 mM NO_x_^–^ 10% LB; row 3, 1 mM NO_x_^–^ 100% LB; and row 4, 1 mM NO_x_^–^ 10% LB. Each plot depicts DNRN and denitrification rates for one biological replicate over time. Representative plots from each condition are shown for brevity.

**Supplementary Figure 6: Time series data under 10 mM NO_x_^–^ and 100% LB conditions (high carbon and NO_x_^–^) for a,b** 10:0 NO_3_^–^/NO_2_^–^ ratios for the second run with new inocula **c,d** 9:1 NO_3_^–^/NO_2_^–^ ratios **e,f** 5:5 NO_3_^–^/NO_2_^–^ ratios and **g,h** 1:9 NO_3_^–^/NO_2_^–^ ratios. 10:0 ratios for the first run are in Figure 2. NO_x_^–^ concentrations (green), NO_2_^–^ (purple), and bacterial growth (black) are shown. Data points are the means of two biological replicates per condition, with error bars indicating ranges. Lefthand panels show WT; righthand show the ∆+∆ co-culture. Curves for NO_x_^–^ and NO_2_^–^ were smoothed with a Savitsky-Golay filter, while OD_600_ curves were fit to a logistic growth model. The R^2^ values indicate the goodness of fit between the logistic curve and the data.

**Supplementary Figure 7: Time series data under 10 mM NO_x_^–^ and 10% LB conditions (low carbon) for a,b** 10:0 NO_3_^–^/NO_2_^–^ ratios for the second run with new inocula **c,d** 9:1 NO_3_^–^/NO_2_^–^ ratios **e,f** 5:5 NO_3_^–^/NO_2_^–^ ratios and **g,h** 1:9 NO_3_^–^/NO_2_^–^ ratios. 10:0 ratios for the first run are in Figure 2. ­­NO_x_^–^ concentrations (green), NO_2_^–^ (purple), and bacterial growth (black) are shown. Data points are the means of two biological replicates per condition, with error bars indicating ranges. Lefthand panels show WT; righthand show the ∆+∆ co-culture. Curves for NO_x_^–^ and NO_2_^–^ were smoothed with a Savitsky-Golay filter, while OD_600_ curves were fit to a logistic growth model. The R^2^ values indicate the goodness of fit between the logistic curve and the data. ­­­

**Supplementary Figure 8: Time series data under 1 mM NO_x_^–^ and 100% LB conditions (low NO_x_^–^) for a,b** 10:0 NO_3_^–^/NO_2_^–^ ratios for the second run with new inocula **c,d** 9:1 NO_3_^–^/NO_2_^–^ ratios **e,f** 5:5 NO_3_^–^/NO_2_^–^ ratios and **g,h** 1:9 NO_3_^–^/NO_2_^–^ ratios. 10:0 ratios for the first run are in Figure 2. NO_x_^–^ concentrations (green), NO_2_^–^ (purple), and bacterial growth (black) are shown. Data points are the means of two biological replicates per condition, with error bars indicating ranges. Lefthand panels show WT; righthand show the ∆+∆ co-culture. Curves for NO_x_^–^ and NO_2_^–^ were smoothed with a Savitsky-Golay filter, while OD_600_ curves were fit to a logistic growth model. The R^2^ values indicate the goodness of fit between the logistic curve and the data.

**Supplementary Figure 9: Time series data under 1 mM NO_x_^–^ and 10% LB conditions (low carbon and NO_x_^–^) for a,b** 10:0 NO_3_^–^/NO_2_^–^ ratios for the second run with new inocula **c,d** 9:1 NO_3_^–^/NO_2_^–^ ratios **e,f** 5:5 NO_3_^–^/NO_2_^–^ ratios and **g,h** 1:9 NO_3_^–^/NO_2_^–^ ratios. 10:0 ratios for the first run are in Figure 2. NO_x_^–^ concentrations (green), NO_2_^–^ (purple), and bacterial growth (black) are shown. Data points are the means of two biological replicates per condition, with error bars indicating ranges. Lefthand panels show WT; righthand show the ∆+∆ co-culture. Curves for NO_x_^–^ and NO_2_^–^ were smoothed with a Savitsky-Golay filter, while OD_600_ curves were fit to a logistic growth model. The R^2^ values indicate the goodness of fit between the logistic curve and the data.
